# Ayahuasca Impairs Fear Memory Reconsolidation and Promotes Rapid Structural Plasticity in the Hippocampus and Prelimbic Cortex

**DOI:** 10.1007/s12035-026-06264-2

**Published:** 2026-10-12

**Authors:** Daiane Momo Daneluz, Alana Sueli dos Santos, Silvana Regina de Melo, Rúbia Maria Weffort de Oliveira, Cristina Aparecida Jark Stern

**Affiliations:** 1 Department of Pharmacology, Federal University of Paraná, Curitiba, PR Brazil; 2 Department of Biochemistry and Molecular Biology, Federal University of Paraná, Curitiba, PR Brazil; 3 Department of Morphology Sciences, State University of Maringá, Maringá, Paraná Brazil; 4 Department of Pharmacology and Therapeutics, State University of Maringá, Maringá, Paraná Brazil

**Keywords:** Psychedelics, Neuroplasticity, Dendritic spines, DMT, Fear conditioning

## Abstract

Psychedelic drugs promote rapid neuroplasticity in brain regions affected by trauma exposure, such as the prelimbic (PL) cortex and the dorsal hippocampus (DH), being investigated as potential treatments for posttraumatic stress disorder. Ayahuasca (Aya) contains N,N-dimethyltryptamine, a classic psychedelic. Our previous work showed that Aya reduces fear expression by impairing fear memory reconsolidation. We hypothesized that Aya administration during reconsolidation induces neuroplastic changes in DH and PL cortex associated with its behavioral effects. Adult male Wistar rats underwent contextual fear conditioning and received Aya (60 mg/kg, oral gavage) or H₂O (vehicle) 20 min before or 3 h after fear memory retrieval. To confirm the reconsolidation-impairing effect of Aya, animals were re-exposed to the conditioned context 24 h later. Structural plasticity was assessed 24 h after retrieval and drug administration by Golgi-Cox staining of the DH and the PL cortex. Neurogenesis was evaluated 10 days later by doublecortin (DCX) immunohistochemistry of dentate gyrus. Aya significantly reduced freezing behavior, confirming the reconsolidation-impairing effect. This outcome was accompanied by increased dendritic spine density in the CA1, CA3, and dentate gyrus regions of the DH, as well as in the PL cortex. In the PL cortex, Aya altered dendritic branching and length depending on the timing of administration. In contrast, Aya did not affect adult hippocampal neurogenesis. Together, these findings suggest that rapid synaptic remodeling in the DH and PL cortex, rather than changes in adult hippocampal neurogenesis, is associated with the reconsolidation-impairing effect of Aya.

**Graphical Abstract:**

**Figure:**
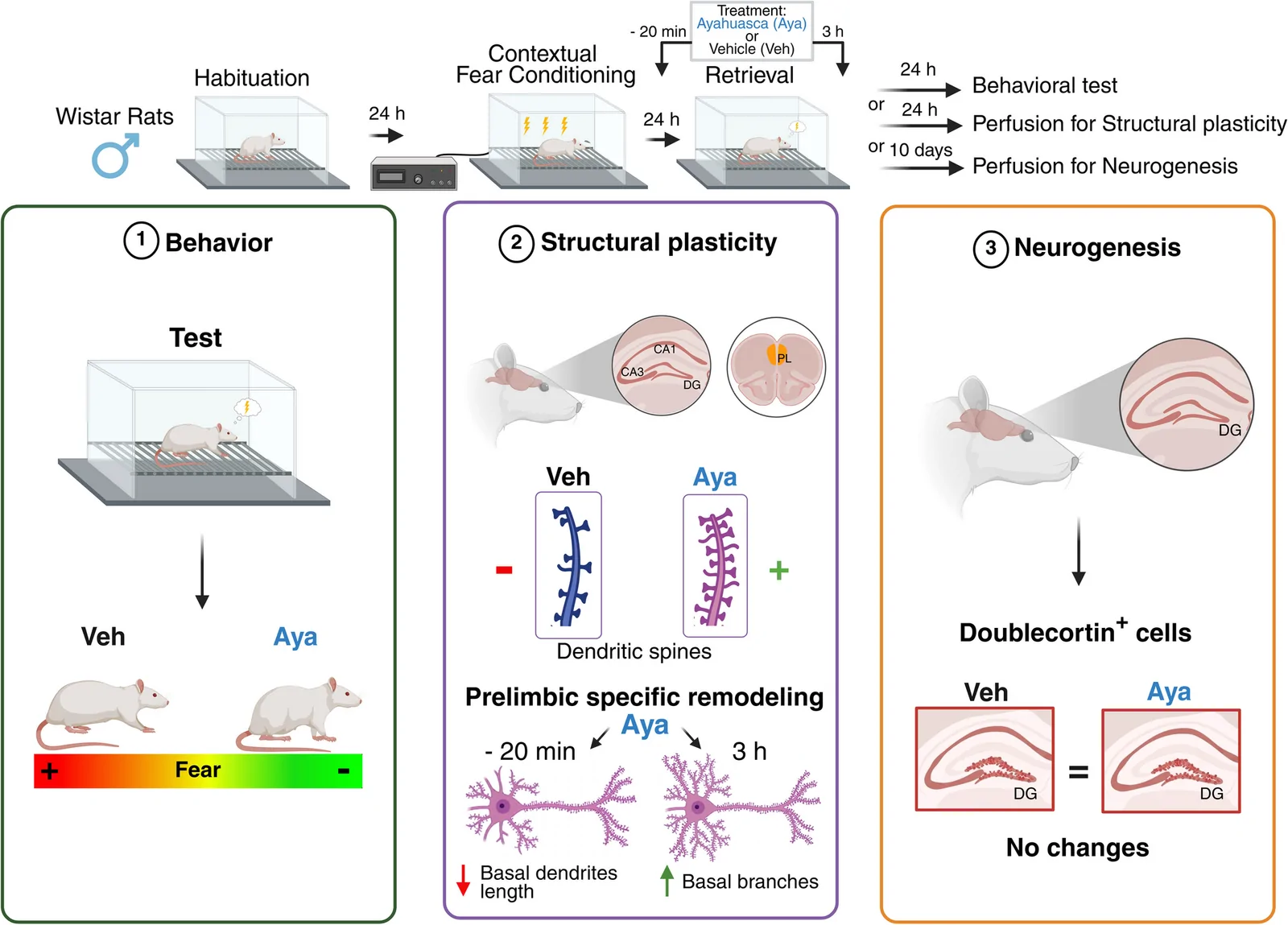

## Introduction

Post-traumatic stress disorder (PTSD) is a stress-related disorder, in which aversive memories play a central role [1, 2]. Converging evidence from humans and animal models shows that trauma and stress might reshape and reduce connectivity in areas related to emotional processing and memory such as medial prefrontal cortex (mPFC) and the hippocampus [3–11]. In this context, psychedelic drugs have been studied for the treatment of PTSD, among other purposes, due to their ability to promote rapid neuroplasticity in the brain areas affected by PTSD [12, 13].

Memory reconsolidation is a transient period triggered after retrieval of a consolidated memory where it becomes labile and susceptible to modification during a time-window that seems to change according to the involved mechanism [14]. We demonstrated that Ayahuasca (Aya), a psychedelic brew, impairs fear memory reconsolidation when administered before or 3 h after retrieval [15]. Aya contains the psychedelic compound N,N-dimethyltryptamine (DMT) and β-carbolines, such as harmine, harmaline, tetrahydroharmine, which are MAO-A inhibitors, allowing DMT to reach the systemic circulation and the brain after oral ingestion [16].

One of the mechanisms that may underlie the reconsolidation impairing effect of Aya is the modulation of neuroplasticity in hippocampal subregions like the dentate gyrus (DG) and the Cornu Ammonis areas CA1 and CA3, as well as the prelimbic (PL) region of the medial prefrontal cortex (mPFC), which are involved in the retrieval, destabilization, and reconsolidation of fear memories [13, 17–21].

Accordingly, Comas Mutis and colleagues demonstrated that memory destabilization and reconsolidation are accompanied by temporally regulated changes in dendritic spine density in the CA1 region of the dorsal hippocampus (DH) [22]. Specifically, dendritic spine density was significantly reduced at 60 min post-retrieval, returning to near-baseline levels after 24 h, suggesting that structural plasticity is tightly coupled with memory updating processes. Moreover, pharmacological blockade of destabilization or reconsolidation mechanisms altered this spine remodeling dynamics, reinforcing the notion that reconsolidation requires active restructuring of synaptic connectivity. These observations are in line with studies showing that long-lasting memories are associated with populations of stable dendritic spines, whereas memory updating and behavioral adaptation involve selective spine formation and elimination [23–25]. Beyond structural plasticity, evidence suggests that adult hippocampal neurogenesis also contributes to reorganizing memory during remote memory reconsolidation [26–28]. For instance, silencing neurogenesis during learning impaired retrieval and remote memory reconsolidation [27]. The adult-born neurons in the dentate gyrus (DG) exhibit high excitability and synaptic plasticity, contributing to contextual discrimination, cognitive flexibility, and emotional memory processing [29–32].

DMT, the main active compound in Aya, enhances structural plasticity in cortical cells [33]. A single dose of DMT significantly increased both basal and apical dendritic spines in rats in vivo [33]. In addition, DMT has been demonstrated to induce hippocampal neurogenesis in mice both in vivo and in vitro [34, 35]. Although substantial evidence supports the capacity of psychedelics to enhance neuroplasticity, it remains unknown whether Aya modulates neuroplastic processes when administered during fear memory reconsolidation. Here, we hypothesized that Aya administration at early and late points within the reconsolidation window would elicit distinct neuroplasticity and neurogenesis changes, revealing potential mechanisms underlying Aya's effects on fear memory processing.

To address this, we used contextual fear conditioning to investigate the impact of Aya given to rats 20 min before or 3 h after retrieval on early and late time points of reconsolidation. Then, through Golgi-Cox staining, we assessed the effects of Aya on dendritic remodeling in CA1, CA3, DG of the DH and in the PL. To assess the involvement of neurogenesis, we also performed immunohistochemistry to evaluate doublecortin (DCX) expression [31].

## Materials and Methods

### Animals

Adult male Wistar rats (3 months old) obtained from the Biological Sciences Sector of the Federal University of Paraná were used. They were housed in polycarbonate cages (48 × 37.5 × 21 cm) in groups of four per cage, with free access to food and water. They were maintained under controlled environmental conditions, with a room temperature of 22 ± 2 °C and a 12-h light/dark cycle (lights on from 7:00 a.m. to 7:00 p.m.). All procedures were approved by the Local Ethics Committee (authorization number 1572) and conducted in accordance with Brazilian regulations.

### Drugs

The Aya brew was donated by Igreja União do Vegetal (Curitiba, Paraná, Brazil). Aya was lyophilized and chemically characterized as previously described [15]. Aya 60 mg/kg was diluted in 2.2 mL of tap water, which served as the vehicle control (Veh). This dose was selected based on our previous work demonstrating that 60 mg/kg of Aya impaired fear memory reconsolidation in rats [15]. According to our previous work, the main Aya constituents of the dosage used here were as follows: DMT, 0.24 mg; tetrahydroharmaline, 0.41 mg; harmaline, 0.06 mg; and harmine, 0.38 mg [15]. Given that Aya is consumed orally in ritual contexts and in accordance with prior experimental studies, all animals received Aya or Veh via oral gavage (o.g.) [15, 36, 37].

### Contextual Fear Conditioning

Contextual fear conditioning was performed in a rectangular chamber (35 × 20 × 30 cm) with aluminum walls and a transparent Plexiglas front wall and ceiling. The grid floor consisted of stainless-steel bars connected to a shock generator (Insight, Ribeirão Preto, Brazil) for electrical foot shock delivery.

Before the beginning of the experimental procedures, rats were randomly assigned to their respective treatment groups. According to previous works, animals were individually placed in the chamber for a 3 min habituation session and were allowed to explore the context [15, 38]. This session was intended to habituate the animals with the spatial and sensory features of the context before subsequent fear conditioning. Following habituation, rats were returned to their home cages. Twenty-four hours later, animals were exposed to the same context and underwent fear conditioning. After 30 s of context exposure, the unconditioned stimulus (US) was delivered as three consecutive foot shocks (0.7 mA, 3 s each, 30 s inter-shock interval). Rats remained in the chamber for 30 s after the last shock and then returned to their home cages. The following day, rats were re-exposed to the conditioned context for 3 min to assess memory retrieval and induce reconsolidation. Twenty min before or 3 h after retrieval each cohort of animals received Aya or Veh according to the previously assigned treatment group. Drug administration was performed in a separate room from both the animal housing facility and the behavioral testing room. The next day, rats underwent a 3 min exposure to the conditioned context (Test) to evaluate the effect of treatment. Freezing behavior was recorded by a trained observer blinded to group assignments and served as an index of fear memory. Independent groups of animals were transcardially perfused at different time points: animals were perfused 1 day after treatment for the analysis of structural plasticity, whereas separate cohorts were perfused 10 days after treatment for the analysis of neurogenesis.

### Golgi–Cox Staining

Golgi-Cox staining was based on previous studies with some modifications [39–41]. Twenty-four hours after retrieval and treatment, rats were deeply anesthetized with 1.0 ml of a solution containing chloral hydrate (2.3 mg/mL, Vetec) and xylazine (10 mg/ml) and transcardially perfused with 0.9% saline containing 0.1 M phosphate buffer (pH 7.4) [42–45]. Brains were immediately collected and immersed in freshly prepared Golgi-Cox solution for 24 h at 37 °C, then transferred to fresh Golgi-Cox solution and kept for approximately 19 days in the dark at room temperature. Brains were subsequently transferred to 30% sucrose solution for cryoprotection, frozen on dry ice and stored at − 80 °C until sectioning.

Coronal Sects. (100 µm) from medial prefrontal cortex (mPFC) and dorsal hippocampus (DH) corresponding to stereotaxic coordinates + 3.8 mm to + 2.2 mm and −3.15 to −4.6 mm from Bregma respectively [46], were obtained using a cryostat (Criocut 1800, Reichert-Jung, Heidelberg, Germany) and mounted onto gelatin-coated slides, that were kept in darkness for 12–24 h before impregnation. All impregnation steps were performed under low-light conditions as follows: distilled water (1 min), ammonium solution (40 min), distilled water (5 min), Rapid Fix (40 min), distilled water (2 × 1 min), ethanol 50% (1 min), 70% (1 min), 95% (1 min), 100% (3 × 5 min), xylene I (10 min), and xylene II (10 min). Slides were coverslipped immediately to avoid xylene evaporation and stored in the dark until imaging.

Pyramidal neurons from layers II/III of the PL cortex, pyramidal neurons from hippocampal subfields CA1, CA3 and granular neurons from dentate gyrus (DG) were imaged using a Leica DM 2500 microscope with a Leica DF345FX camera. The criteria used to select the neurons to be analyzed included: relatively isolated neurons with a clearly defined soma, complete whole-cell impregnation, and well-distinguished dendritic arborization [39]. For each animal, five neurons per region were analyzed; from each neuron, three basal dendritic branches and one apical branch were photographed for quantification of dendritic spine density. Spine density was quantified manually along a 30 µm segment of each dendritic branch using Fiji/ImageJ. Neuronal morphology was assessed using the Simple Neurite Tracer plugin (SNT, version 4.2.1) in FIJI; dendrites were manually traced to determine the number of neurites and total dendritic length per neuron. All analyses were performed by investigators blinded to the experimental group assignments.

### Immunohistochemistry

The immunohistochemistry protocol was adapted from previously described methods [47]. Ten days after retrieval and treatment, rats were deeply anesthetized and transcardially perfused with 0.01 M phosphate-buffered saline (PBS) for 20 min at a flow rate of 10 mL/min, followed by 4% paraformaldehyde (PFA) in 0.2 M phosphate buffer (PB) for an additional 20 min at the same flow rate. Brains were post-fixed in PFA for 12 h and subsequently cryoprotected by sequential immersion in 20% and 30% sucrose solutions prepared in 0.01 M PBS for 24 h each at 4 °C. Brains were then rapidly frozen on dry ice and stored at −80 °C until sectioning.

Coronal Sects. (40 µm) were obtained using a cryostat (Criocut 1800, Reichert-Jung, Heidelberg, Germany). Thirty-six sections spanning the dorsal hippocampus were collected into six alternating tubes containing a cryoprotectant solution (15% sucrose and 30% ethylene glycol in phosphate buffer) and stored at −24 °C until further processing. Sections corresponded to stereotaxic coordinates −3.15 to −4.6 mm from Bregma respectively, according to [46].

Sections were washed in PBS and subjected to antigen retrieval by incubation in a citrate buffer at 50 °C for 30 min, followed by additional PBS washes. Endogenous peroxidase activity was quenched for 30 min, and non-specific binding was blocked with 5% bovine serum albumin (BSA) in PBS for 1 h at room temperature. Sections were then incubated overnight at 4 °C with the primary antibody anti-Doublecortin (DCX; Cell Signaling #4604, polyclonal rabbit, 1:1000) diluted in PBS containing 0.3% Triton X-100. After PBS washes, sections were incubated with a biotinylated secondary antibody (mouse anti-rabbit IgG; Santa Cruz #sc-2491, 1:500) for 2 h at room temperature. Signal amplification was performed using the Vectastain ABC kit (Vector Laboratories) for 2 h, followed by chromogenic development with nickel-enhanced diaminobenzidine (NiDAB) for 10 min. Sections were mounted onto gelatin-coated slides, air-dried for 7 days, cleared in xylene, and coverslipped with Permount® (Fisher Scientific, Fair Lawn, NJ, USA).

Quantitative analysis was performed using a Leica DM2500 microscope equipped with a Leica DF345 FX camera. DCX-immunoreactive (DCX-IR) neurons were manually counted bilaterally throughout the DG. DG area was measured using ImageJ, and cell density was expressed as the number of DCX-IR cells per unit area (cells/mm^2^). Data are presented as mean ± SEM from 3–4 sections per animal. All analyses were performed by investigators blinded to experimental group assignments.

### Statistics

Sample sizes were determined using G-Power (HHU: Universität Düsseldorf, Germany), with a minimun of 5 rats per group for Golgi-cox staining and immunostaining and a minimum of 8 rats for behavioral tests. Due to some issues during the staining process, the staining was not consistent in some samples, therefore they were excluded from the analysis. After testing for normality using Shapiro–Wilk test and homogeneity of variances using Levene’s test, statistical analysis was performed using unpaired Student’s t-test when assumptions of normality and equal variances were met; otherwise, two-tailed Welch t-test was applied. For comparisons involving more than two groups we used one-way or two-way repeated measures ANOVA. The Tukey test was used for post-hoc comparisons (*p* < 0.05). Outliers were identified using the Grubbs test. Effect sizes were estimated using Cohen’s *d* for two-group comparisons and eta-squared (η^2^) for ANOVA analyses [48]. Results were considered statistically significant at *p* < 0.05 and are presented as mean ± SEM. Statistical analysis was performed with Statistic 12 (StatSoft, USA) and GraphPad Prism 11 (GraphPad Prism, USA) was used for graphing.

### Experimental Design

For all experiments, rats underwent contextual fear conditioning followed by memory retrieval. Animals were randomly assigned to one of four conditioned groups according to treatment timing and treatment: Aya or Veh administered either 20 min before or 3 h after memory retrieval. The effects of treatment were subsequently assessed at different time points and using distinct experimental endpoints.

**Fig. 1:**
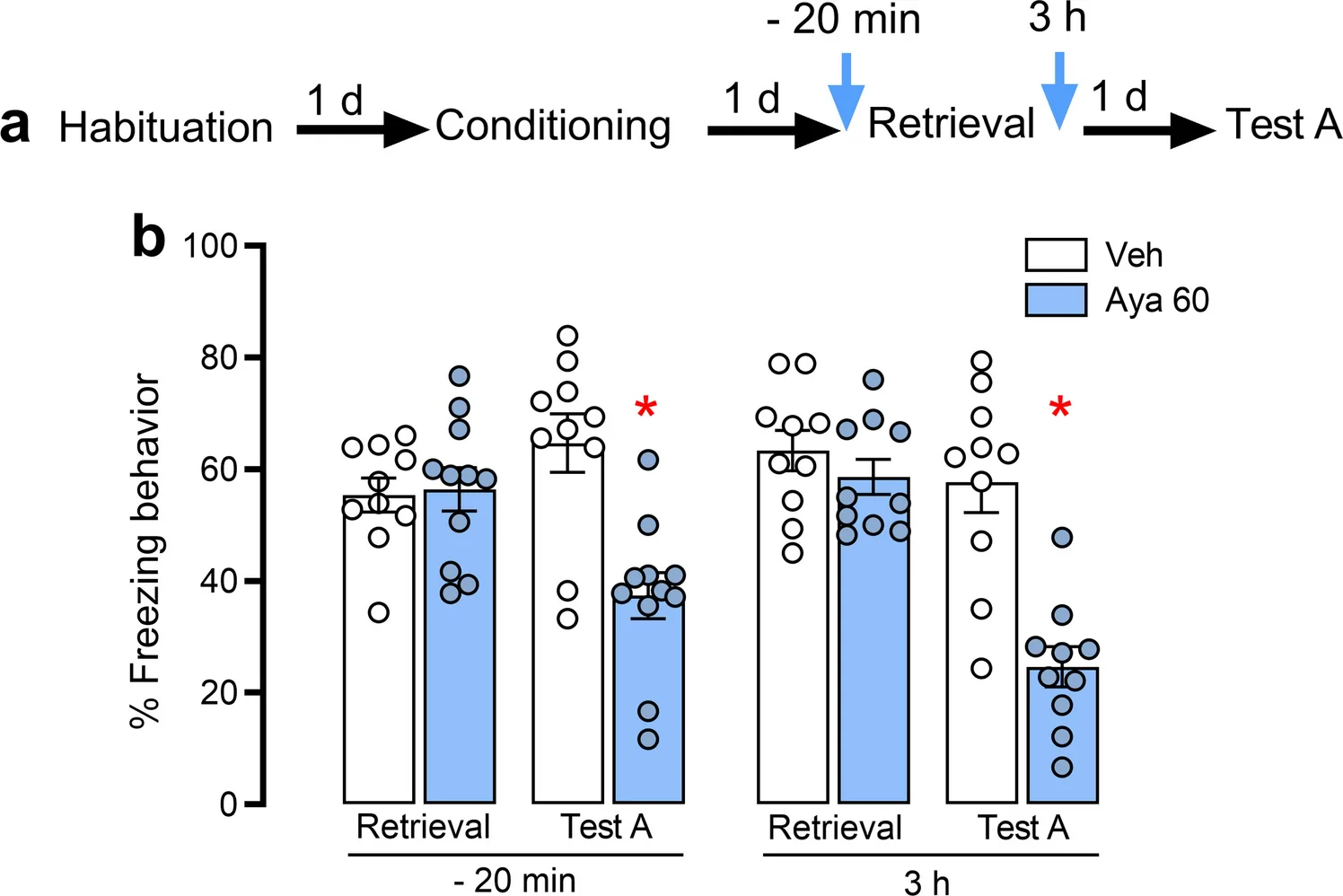
Effects of Aya treatment 20 min before or 3 h after fear memory retrieval. **a**: Experimental timeline. **b**: Bar graphs show the mean percentage of freezing behavior of Veh-treated and Aya-treated animals during retrieval and Test A. Groups are organized according to the timing of treatment relative to memory retrieval (20 min before and 3 h after memory retrieval). Data are presented as mean ± SEM and individual values. The * indicates *p* < 0.05 compared with the session control group. Number of animals per group:—20 min: Veh = 10; Aya = 11; 3 h: Veh = 10; Aya = 10

In Experiment 1, we evaluated the effect of Aya on fear memory reconsolidation. Forty-one rats were assigned to one of the four conditioned groups described above. Fear memory was assessed 24 h after treatment during the Test A (Fig. 1a). No brain tissue was collected from this cohort.

**Fig. 2:**
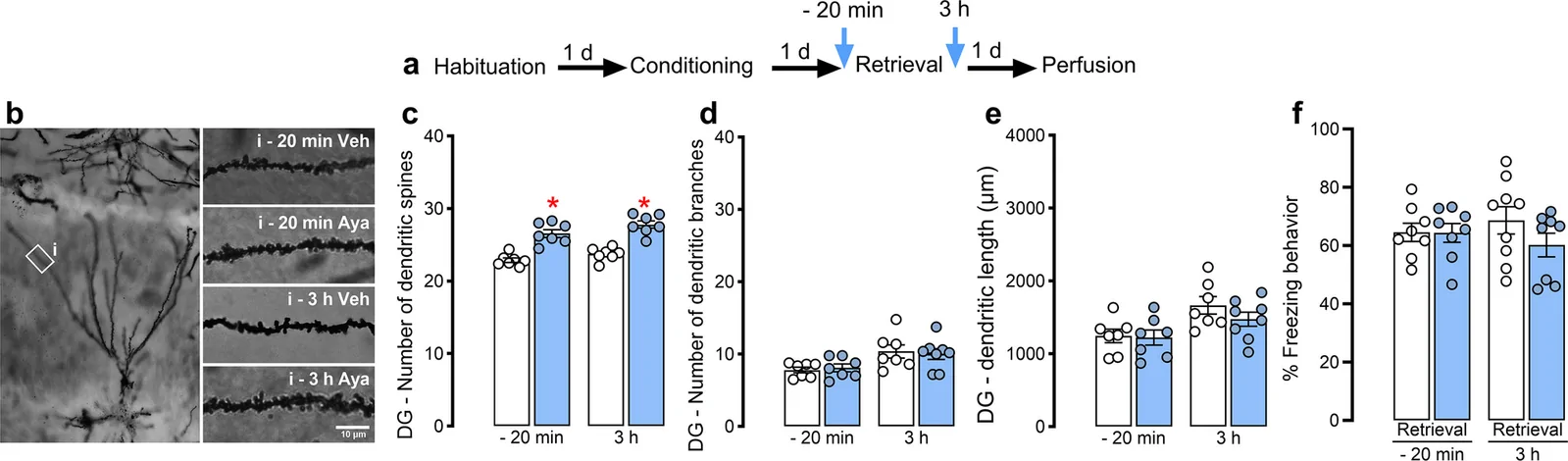
Effects of Aya treatment on DG dendritic spines. **a**: Experimental timeline. **b**: Representative 20 × microphotograph of DG granule neuron; **i**: representative 100 × microphotographs of segments of groups treated with Veh or Aya. **c**: DG dendritic spine density: Bar graphs show the mean number of spines per 30 µm segment in Veh- and Aya-treated animals, grouped by treatment timing relative to memory retrieval (20 min before or 3 h after). **d**: DG dendritic branching: Bar graphs represent the mean number of basal and apical dendritic branches per neuron in Veh- and Aya-treated animals, grouped by treatment timing. **e**: DG dendritic length: Bar graphs show the length mean of basal and apical dendrites in Veh- and Aya-treated animals, grouped by treatment timing. **f**: Bar graphs show the mean percentage of freezing behavior of Veh-treated and Aya-treated animals during retrieval. Groups are organized according to the timing of treatment relative to memory retrieval (20 min before and 3 h after memory retrieval). Data are presented as mean ± SEM and individual values. The * indicates *p* < 0.05 compared with the session control group. Number of animals per group: -20 min: Veh = 7–8; Aya = 7–8; 3 h: Veh = 9; Aya = 8

**Fig. 3:**
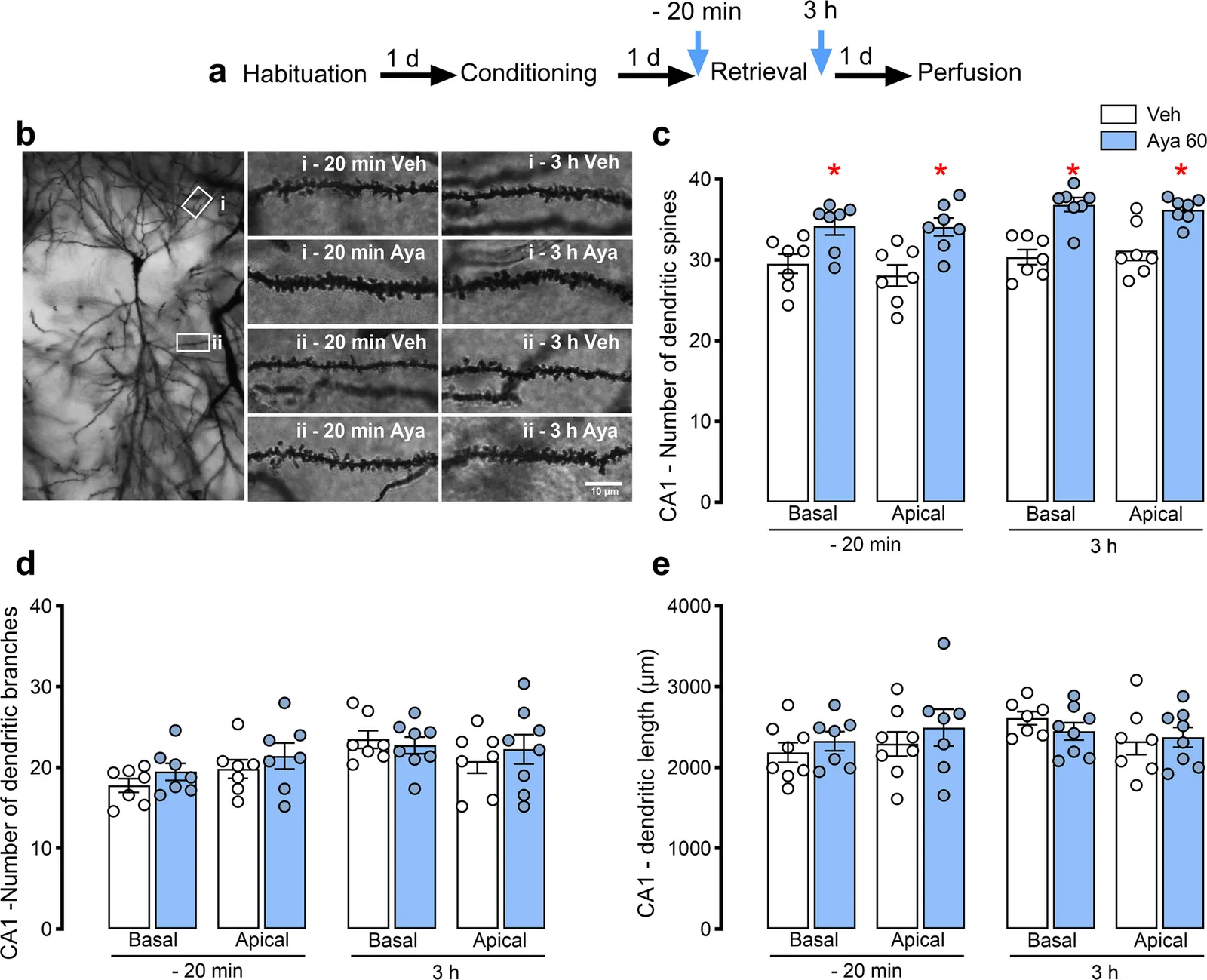
Effects of Aya treatment on CA1 dendritic spines. **a**: Experimental timeline. **b**: Representative 20 × microphotograph of a CA1 pyramidal neuron; **i**: representative 100 × microphotographs of basal segments of groups treated with Veh or Aya. **ii**: representative 100 × microphotographs of apical segments of groups treated with Veh or Aya. **c**: CA1 dendritic spine density: Bar graphs show the mean number of spines per 30 µm segment of basal and apical dendrites in Veh- and Aya-treated animals, grouped by treatment timing relative to memory retrieval (20 min before or 3 h after). **d**: CA1 dendritic branching: Bar graphs represent the mean number of basal and apical dendritic branches per neuron in Veh- and Aya-treated animals, grouped by treatment timing. **e**: CA1 dendritic length: Bar graphs show the mean of the length of basal and apical dendrites in Veh- and Aya-treated animals, grouped by treatment timing. Data are presented as mean ± SEM and individual values. The * indicates *p* < 0.05 compared with the session control group. Number of animals per group: -20 min: Veh = 7; Aya = 7; 3 h: Veh = 7; Aya = 8

**Fig. 4:**
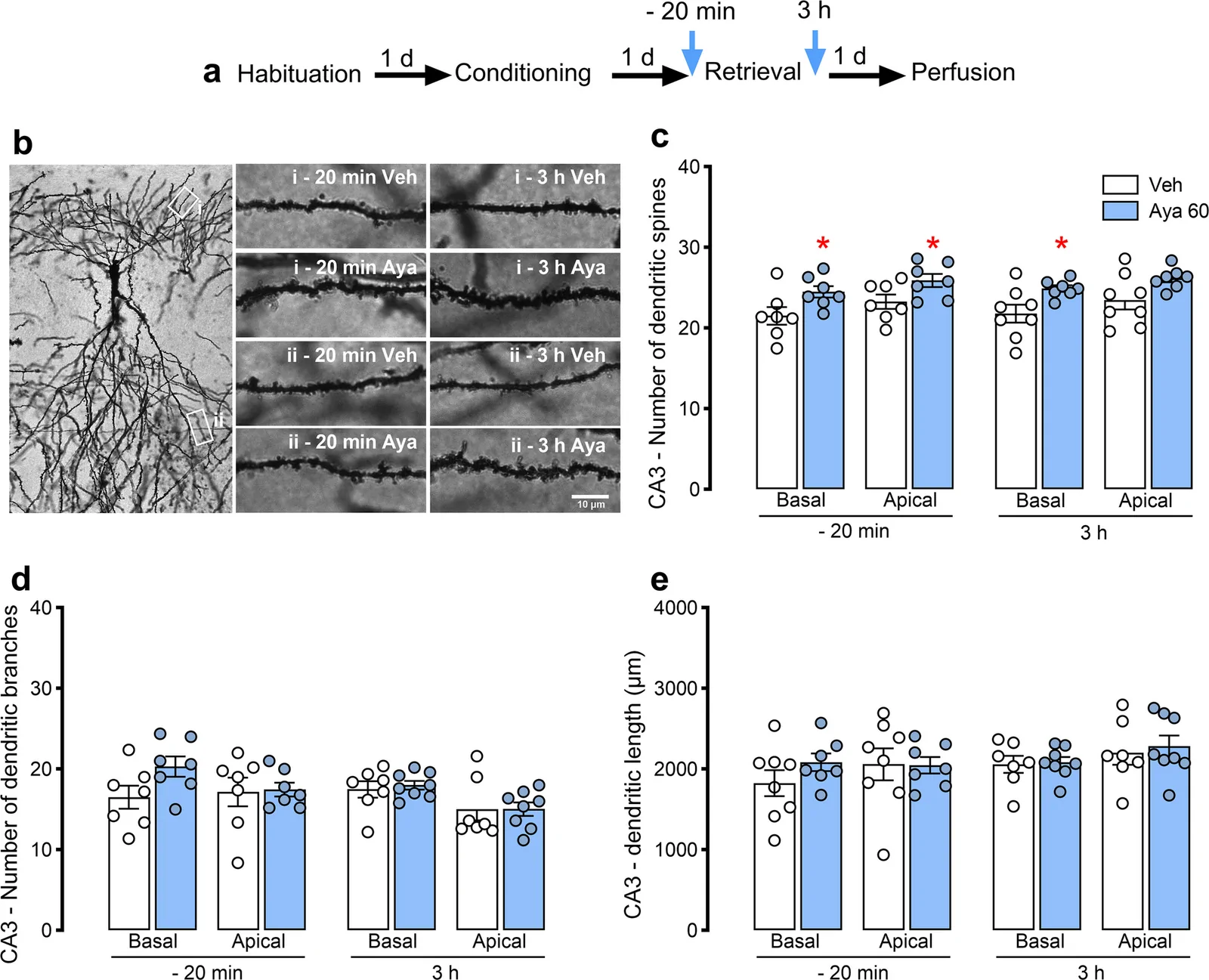
Effects of Aya treatment on CA3 dendritic spines. **a**: Experimental timeline. **b**: Representative 20 × microphotograph of a CA3 pyramidal neuron; **i**: representative 100 × microphotographs of basal segments of groups treated with Veh or Aya. **ii**: representative 100 × microphotographs of apical segments of groups treated with Veh or Aya. **c**: CA3 dendritic spine density: Bar graphs show the mean number of spines per 30 µm segment of basal and apical dendrites in Veh- and Aya-treated animals, grouped by treatment timing relative to memory retrieval (20 min before or 3 h after). **d**: CA3 dendritic branching: Bar graphs represent the mean number of basal and apical dendritic branches per neuron in Veh- and Aya-treated animals, grouped by treatment timing. **e**: CA3 dendritic length: Bar graphs show the mean of the length of basal and apical dendrites in Veh- and Aya-treated animals, grouped by treatment timing. Data are presented as mean ± SEM and individual values. The * indicates *p* < 0.05 compared with the session control group. Number of animals per group: -20 min: Veh = 7; Aya = 7; 3 h: Veh = 7; Aya = 8

**Fig. 5:**
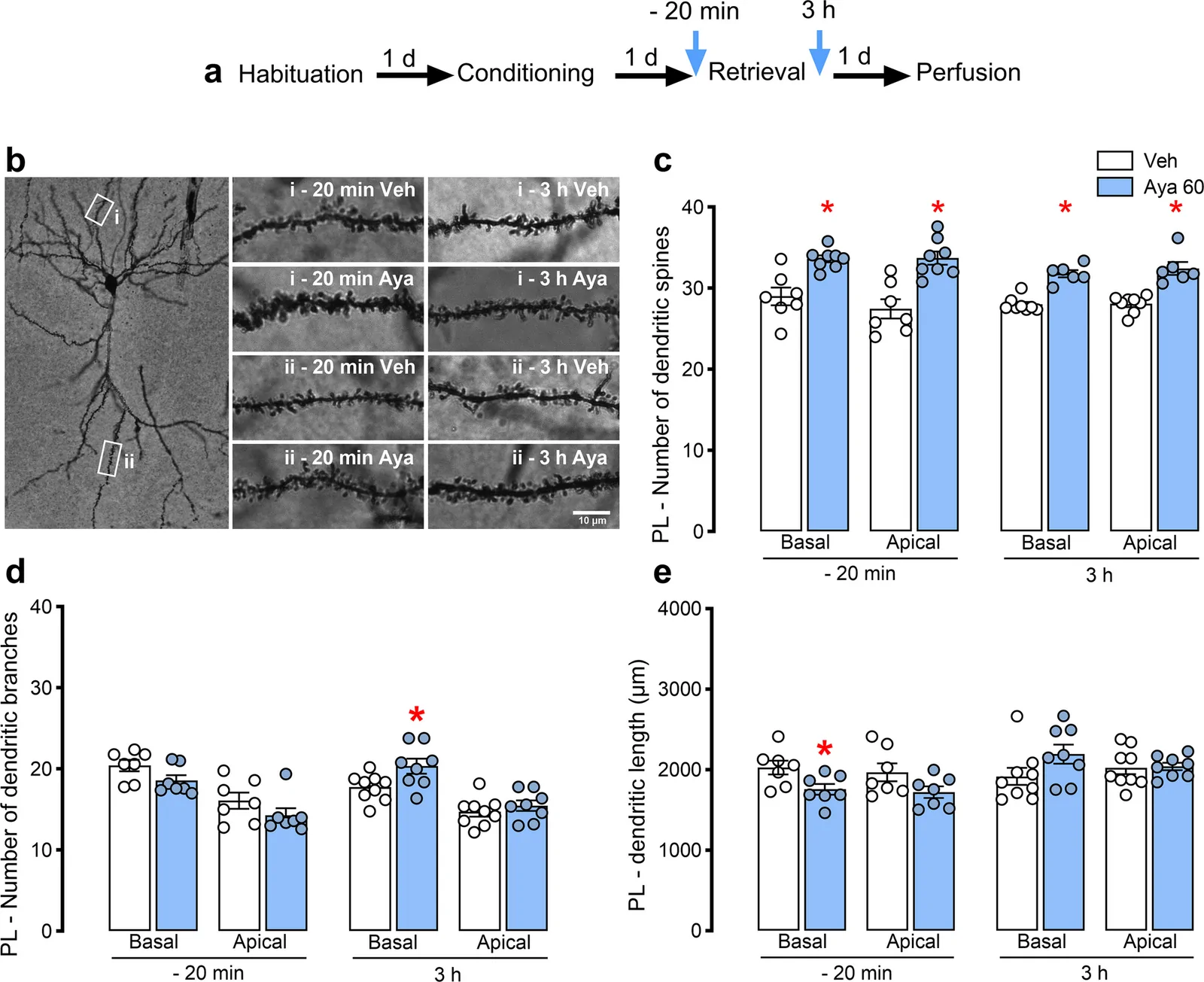
Effects of Aya treatment on PL dendritic spines. **a**: Experimental timeline. **b**: Representative 20 × microphotograph of a PL pyramidal neuron; **i**: representative 100 × microphotographs of basal segments of groups treated with Veh or Aya. **ii**: representative 100 × microphotographs of apical segments of groups treated with Veh or Aya. **c**: PL dendritic spine density: Bar graphs show the mean number of spines per 30 µm segment of basal and apical dendrites in Veh- and Aya-treated animals, grouped by treatment timing relative to memory retrieval (20 min before or 3 h after). **d**: PL dendritic branching: Bar graphs represent the mean number of basal and apical dendritic branches per neuron in Veh- and Aya-treated animals, grouped by treatment timing. **e**: PL dendritic length: Bar graphs show the mean of the length of basal and apical dendrites in Veh- and Aya-treated animals, grouped by treatment timing. Data are presented as mean ± SEM and individual values. The * indicates *p* < 0.05 compared with the session control group. Number of animals per group: -20 min: Veh = 7; Aya = 7; 3 h: Veh = 9; Aya = 8

In Experiment 2, we evaluate whether the behavioral effect of A observed when administered at different time points relative to retrieval was associated with changes in structural plasticity. One day after treatment, thirty-four rats from independent groups were transcardially perfused, and brains were collected for Golgi-Cox staining (Figs. 2–5a).

**Fig. 6:**
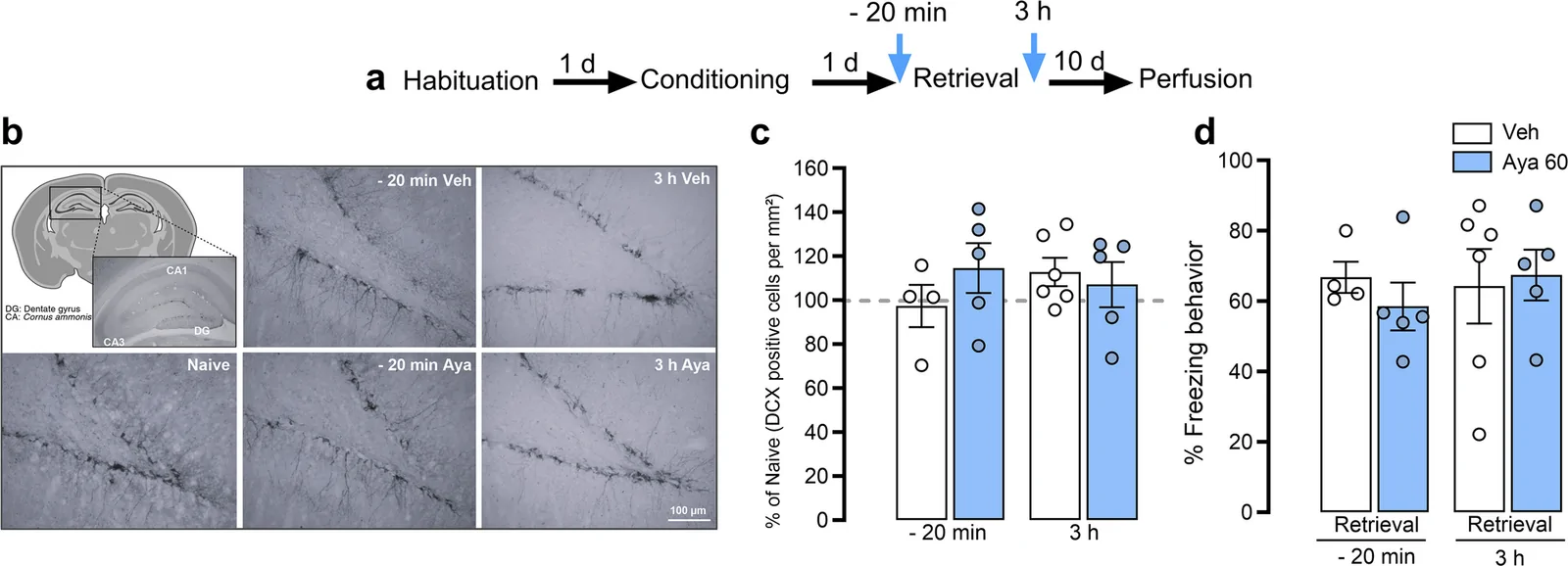
Effects of Aya treatment DCX expression in DG. **a**: Experimental timeline. **b**: The top image on the left represents a stained hippocampus, followed by representative 20 × microphotographs of naïve animals or groups treated with Veh or Aya 20 min before or 3 h after retrieval. **c**: Data are expressed as a percentage of the mean number of DCX positive cells per mm^2^ in the dentate gyrus of the naive group for visualization purposes, with the naive group represented as a dashed reference line at 100%. **d**: Bar graphs show the mean percentage of freezing behavior of Veh-treated and Aya-treated animals during retrieval. Groups are organized according to the timing of treatment relative to memory retrieval (20 min before and 3 h after memory retrieval). Data are presented as mean ± SEM and individual values. The * indicates *p* < 0.05 compared with the session control group. Number of animals per group: Naive: 6; -20 min: Veh = 4; Aya = 5; 3 h: Veh = 6; Aya = 5

In Experiment 3, we evaluate the effect of Aya administration on neurogenesis. To determine whether the persistent behavioral effects of Aya could be accompanied by changes in the population of immature hippocampal neurons, ten days after treatment, twenty rats from independent groups, and an additional naive group, which did not undergo conditioning or receive treatment, were transcardially perfused. Brains were collected for immunohistochemical analysis of doublecortin (DCX) expression (Fig. 6a).

## Results

### Result 1: Aya Treatment Impairs Fear Memory Reconsolidation

Aya treatment 20 min before memory retrieval or 3 h later impaired fear memory reconsolidation. Two-way repeated measures ANOVA revealed significant main effects of treatment (F_1,37_ = 25.5, *p* = 0.00001; η2 = 0.41) and repetition (F_1,37_ = 22.4, *p* = 0.00003; η2 = 0.37) and no significant main effects of time of treatment (F_1,37_ = 0.56, *p* = 0.46; η2 = 0.14). No interaction among treatment, repetition and time of treatment was detected (F_1,37_ = 0,00013; *p* = 0.99; η2 = 0.000004), however, a significant interaction between repetition and treatment (F_1,37_ = 29,3; *p* = 0.000001; η2 = 0.44) and repetition and time of treatment (F_1,37_ = 8,2; *p* = 0.007; η2 = 0.18) were detected. Tukey’s post hoc analysis of treatment and repetition interaction showed that Aya treatment significantly reduced freezing behavior in both moments (Fig. 1b). Together, the results suggest that Aya treatment impaired fear memory reconsolidation, regardless of the moment of administration.

### Result 2: Aya Treatment During Reconsolidation Changes Structural Plasticity in the Dorsal Hippocampus

All groups expressed a similar percentage of freezing behavior during fear memory retrieval (Fig. 2f; -20 min: t_14_ = 0.004, *p* = 0.97, d = 0.022; 3 h: t_15_ = 1.345, *p* = 0.199, d = 0.66).

In the DG neurons, the student’s t test showed that Aya treatment 20 min before or 3 h after retrieval significantly increased the number dendritic spines compared to control (Fig. 2c; -20 min: t_12_ = 5.99, *p* = 0.0001, d = 3.20; 3 h: t_12_ = 6.62, *p* = 0.0001, d = 3.54). No significant differences were observed in the number of dendritic branches or dendritic length of any group and time (Fig. 2d; -20 min: t_12_ = 0.16, *p* = 0.87, d = 0.24; 3 h: t_13_ = 1.25, *p* = 0.23, d = 0.15; Fig. 2e; -20 min: t_12_ = 0.46, *p* = 0.65, d = 0.08; 3 h: t_13_ = 0.30, *p* = 0.767, d = 0.65).

In the CA1 pyramidal neurons, the Student´s t test showed that Aya treatment 20 min before or 3 h after retrieval significantly increased the number of basal (Fig. 3b; -20 min: t_12_ = 2.839, *p* = 0.015, d = 0.68; 3 h: t_12_ = 5.08, *p* = 0.0003, d = 2.71) and apical dendritic spines compared to control (Fig. 3b; -20 min: t_12_ = 3.468, *p* = 0.0047, d = 1.85; 3 h: t_12_ = 3.70, *p* = 0.003, d = 1.98). No significant differences were observed in the number of dendritic branches or dendritic length of any group and time (Fig. 3c-d; Basal -20 min: t_12_ = 1.24, *p* = 0.238, d = 0.43; Apical -20 min: t_12_ = 0.81, *p* = 0.435, d = 0.39; Basal 3 h: t_13_ = 0.47, *p* = 0.645, d = 0.63; Apical 3 h: t_13_ = 0.617, *p* = 0.548, d = 0.13).

In the CA3 pyramidal neurons, the Student´s t test showed that Aya treatment 20 min before significantly increased the number of basal dendritic spines compared to control (Fig. 4c; -20 min: t test, t_12_ = 2.262, *p* = 0.043, d = 1.21; 3 h: t test, t_13_ = 2.511, *p* = 0.026, d = 1.33). A significant enhancement in apical dendritic spines was detected with the treatment 20 min before retrieval (Fig. 4c; -20 min: t_12_ = 2.192, *p* = 0.048, d = 1.17), but not 3 h later (Fig. 4c; Welch’s t test, t_9.61_ = 2.22, *p* = 0.052, d = 1.12). No significant differences were observed in the number of dendritic branches or dendritic length of any group and time (Fig. 4d-e; Basal -20 min: t_12_ = 2.01, *p* = 0.06, d = 0.68; Apical -20 min: t_12_ = 0.14, *p* = 0.88, d = 0.03; Basal 3 h: t_13_ = 0.46, *p* = 0.653, d = 0.1; Apical 3 h: t_13_ = 0.01, *p* = 0.98, d = 0.21). Together, the results suggest that Aya effects on fear memory reconsolidation rely on changes in structural plasticity.

### Result 3: Aya Treatment During Reconsolidation Changes Structural Plasticity in the Prelimbic Cortex

In the PL neurons, the Student's t test showed that Aya treatment 20 min before or 3 h after retrieval significantly increased the number of basal (Fig. 5c; Welch’s t test, -20 min: t_7,82_ = 3.95, *p* = 0.004, d = 2.09; 3 h: t_13_ = 4.49, *p* = 0.0006, d = 3.79) and apical dendritic spines compared to control (Fig. 5c; -20 min: t_13_ = 4.49, *p* = 0.0006, d = 2.29; 3 h: t_12_ = 5.37, *p* = 0.0002, d = 2.76). The Student's t test showed that Aya treatment 3 h after retrieval significantly increased the number of basal dendritic branches compared to control (Fig. 5d; t_15_ = 2.5, *p* = 0.02, d = 1.19), no effect was observed 20 min before retrieval (t_12_ = 1.92, *p* = 0.08, d = 1.03). No significant effects on apical dendritic branches were observed in 3 h (t_15_ = 0.87, *p* = 0.39, d = 0.43) or -20 min (t_12_ = 1.35, *p* = 0.2, d = 1.37) groups. The Student's t test showed that Aya treatment 20 min before retrieval significantly reduced the basal dendritic length compared to control (Fig. 5e; t_12_ = 2.46, *p* = 0.03, d = -1.32), no effect was observed 3 h after retrieval (t_15_ = 1.73, *p* = 0.1, d = 0.84). No significant effects were observed in apical dendritic length (-20 min: t_12_ = 1.86, *p* = 0.09, d = 0.99; 3 h: t_15_ = 0.17, *p* = 0.86, d = 0.08).

### Result 4: Aya Treatment During Reconsolidation Did Not Change Neurogenesis

All groups expressed similar percentage of freezing behavior during fear memory retrieval (Fig. 6d; -20 min: t_7_ = 0.95, *p* = 0.37, d = 0.66; 3 h: t_9_ = 0.238, *p* = 0.81, d = 0.15). In the dentate gyrus one way ANOVA shows that Aya did not change the number of positive DCX neurons when compared to Veh groups either when given 20 min before (F_(2,12)_ = 0.81, *p* = 0.470, η^2^ = 0.118; Fig. 6b) as when given 3 h after retrieval (F_(2, 14)_ = 0.59, *p* = 0.569, η^2^ = 0.07; Fig. 6c).

## Discussion

The present study demonstrates that Aya treatment 20 min before or 3 h after retrieval impairs fear memory reconsolidation, replicating our previous reported findings [15]. This effect was accompanied by a significant increase in dendritic spine density in the CA1, CA3, DG of the DH and PL cortex, observed 24 h after retrieval and treatment regardless of the administration time point. Although Aya promoted structure remodeling of existing neurons, DCX immunostaining showed no evidence of Aya-induced changes of immature neurons at 10 days post-retrieval and treatment, suggesting that the observed behavioral effect and synaptic plasticity happen independently of adult neurogenesis mechanisms.

### Aya Effects on Fear Memory Reconsolidation

The administration of Aya 20 min before or 3 h after retrieval impaired fear memory reconsolidation in male rats. The disruptive effect of Aya on fear memory has been previously reported by our group, but until now rested on a single piece of evidence [15]. The replication of this finding in a new experimental context reinforces the robustness and generalizability of Aya's effect on reconsolidation. These results also support our structural finding of a treatment-induced increase in dendritic spine density, as well as our cellular finding that neurogenesis does not appear to sustain the longer-term effects observed. No other studies reported effects of Aya or its main component, DMT, specifically on reconsolidation. However, other fear memory processes were evaluated under Aya treatment. Studies have shown that repeated administration of Aya facilitates contextual fear memory extinction and reduces fear memory generalization [49, 50]. Thus, indicating that Aya has a broad effect in attenuating the expression of conditioned fear.

The dose and treatment regimen seem to be critical parameters for this attenuating effect. When rats received 120 mg/kg Aya for 30 days prior to fear conditioning, they displayed higher freezing than controls, which is the opposite of the pattern we observe with a smaller and acute dose of Aya given prior or after retrieval [51]. A similar pattern dependent on dose and regimen is observed with DMT alone. The acute treatment with DMT (10 mg/kg) or chronic (1 mg/kg) treatment facilitated cued fear conditioning extinction, but not contextual fear extinction [52, 53]. A recent study showed that low doses of DMT are more effective than high doses in facilitating fear extinction. Here, the 60 mg/kg dose of Aya contains 0.24 mg/kg of DMT, which is approximately three to seven times lower than the amount of DMT contained in the Aya dose used by humans in ritualistic contexts [54, 55].

Other psychedelics, such as phenethylamines like the substituted amphetamine 1-(2,5-dimethoxy-4-iodophenyl)−2-aminopropane (DOI), tryptamines such as psilocybin and entactogens such as 3,4-methylenedioxymethamphetamine (MDMA) can also reduce fear memories through reconsolidation impairment or enhancing fear memory extinction [56–58]. Of note, these psychedelics have been shown to increase dendritic spines in brain areas relevant to modulation of fear memories, such as mPFC and hippocampus [33, 56, 59–61], suggesting that the reshaping of structural plasticity might be one pathway through which psychedelics interfere with reconsolidation processes.

### Aya Effects on Structural Plasticity

Either 20 min before or 3 h post-retrieval, Aya treatment significantly increased dendritic spine density in the CA1, CA3 and DG subfields of the dorsal hippocampus 24 h later, suggesting an enhancement of structural remodeling compared to controls during the reconsolidation window. This finding is consistent with the literature showing that dendritic spines are closely related to memory and learning processes and adds a new dimension regarding the dendritic spine dynamics related to the reconsolidation process [62, 63].

While it is well documented that reconsolidation of fear memories recruits synaptic plasticity mechanisms, the extent to which these changes reflect in dendritic spine modifications has received little direct investigation [17, 23]. Comas Mutis et al. examined pyramidal neurons of CA1 region of the hippocampus of context conditioned rats and showed that 1 h following retrieval into the reconsolidation window, dendritic spine density is transiently reduced, as consequence of memory destabilization, particularly the mature mushroom-shaped spines, with density recovering to the pre-retrieval levels 24 h after retrieval [22].

Complementarily, evidence of in vivo two-photon imaging combined with auditory fear conditioning has shown that retrieval of a remote fear memory (21 days after task acquisition) increased dendritic spines density, mainly mushroom-shaped spines in the DG of the hippocampus, which is interpreted as possibly enhanced afferent synaptic connectivity [64]. Additionally, contextual fear conditioning has been shown to promote structural synaptic plasticity by increasing dendritic spine density and the proportion of thin spines in the ventral hippocampus (VH), while enhanced dendritic spine formation, increased mushroom-shaped and a decrease in the proportion of thin spines in the CA1 region of the DH and VH of mice is observed 6 h after retrieval [65].

The CA1 region of the hippocampus is critical for encoding, retrieval and reconsolidation of fear memories [19, 66, 67]. CA1 receives information from CA3 and from the entorhinal cortex, then transmits the information to cortical and limbic regions involved in emotional regulation, such as prefrontal cortex and amygdala [14, 42, 68]. Thus, the structural reshaping observed 24 h following Aya treatment may reflect synaptic remodeling associated with memory reconsolidation, since we observe an increase in dendritic spine density in different hippocampal subfields. Although Aya administration at both -20 min and 3 h increased dendritic spine density relative to their respective vehicle controls, the magnitude of these effects differed between time points, being higher at 3 h. Importantly, a direct comparison between the Aya-treated groups did not reveal a significant difference in spine density.

Pharmacokinetic evidence indicates that Aya constituents reach the central nervous system and exhibit distinct temporal profiles. DMT is rapidly distributed to the rat brain following intraperitoneal administration, with brain concentrations peaking within approximately 10–15 min and declining rapidly thereafter; an elimination half-life of approximately 5.7 min has been reported for DMT in the rat brain following intraperitoneal administration [69, 70]. However, direct measurements of brain concentrations following oral administration of Aya in rats remain limited. Possibly, differences in pharmacokinetics alone are unlikely to account for the more pronounced effect on CA1 dendritic spine density observed when Aya was administered 3 h after retrieval. Rather, this difference may reflect the dynamic nature of the molecular and cellular processes engaged following memory retrieval and reactivation [14]. In fact, our previous work showing the lack of effect of Aya administered immediately after memory retrieval supports this interpretation [15]. Thus, Aya treatment may produce different effects depending on the stage of memory processing.

Importantly, in the PL region of the mPFC, Aya treatment also induced an increase in dendritic spine density, along with changes in dendrite number and length, 24 h after administration. However, some specific changes depended on treatment timing. When rats received Aya 3 h after retrieval they showed increased basal dendrite branching, whereas those treated 20 min before retrieval showed decreased length of basal segments. This indicates that the timing of Aya administration relative to retrieval differently shapes the morphological remodeling observed 24 h later, even though the behavioral outcome of reduction in freezing behavior was similar across both treatment schedules. Considering that PL cortex activity underlies fear memory reconsolidation and exerts top-down control over emotional and memory processing, the structural remodeling may have contributed to the Aya-induced decrease in fear response [42, 68, 71]. Of note, we analyzed neurons from mPFC layers II/III, which are preferentially innervated by projections from BLA and periaqueductal gray matter [72]. However, differently from the hippocampus, drugs acting at serotonergic transmission enhance cortical structural plasticity also in naive rodents [73].

In the mPFC, the infralimbic cortex (IL) has been implicated in the ability of Aya to modulate fear memories. For instance, Aya facilitates fear extinction through mechanisms involving 5-HT_2A_ and 5-HT_1A_ receptors, whereas BDNF-TrkB signaling has been proposed as a contributor to the neuroplastic effects of Aya in this region [49, 50]. However, few works have examined whether fear extinction and structural plasticity in the IL are directly related. Fear extinction has been associated with changes in dendritic spine density in the IL, although the direction of these changes may depend on the experimental conditions and the timing of analysis. For example, fear extinction was associated with reduced spine density in apical terminal dendrites of IL neurons [74], whereas recent evidence indicates that higher spine density in vmPFC neurons projecting to the BLA is associated with enhanced extinction memory [75]. Thus, structural plasticity in the IL appears to be dynamically associated with extinction-related processes rather than reflecting a uniform increase or decrease in spine density. Considering the complementary roles of the IL and PL in extinction and reconsolidation, respectively, our findings raise the possibility that Aya may engage distinct forms of structural plasticity in these mPFC subregions depending on the memory process being recruited. In our protocol, in which Aya was administered before/after a short retrieval session that engages memory reconsolidation, Aya increased dendritic spine density in the PL. Whether Aya also induces structural changes in IL under extinction-promoting conditions remains undetermined.

Despite producing similar outcomes, i.e., the reduction of freezing behavior, drugs that impair fear memory reconsolidation produced opposite effects on CA1 dendritic spine density 24 h after treatment. For instance, intra-basolateral amygdala (BLA) infusion of propranolol, a nonselective β-adrenergic receptor (β-AR) antagonist immediately after retrieval reduced dendritic spine density, whereas we observed an enhancement. These differences might be regarding different mechanisms of action of each compound, but may also reflect methodological differences, including the route of administration (oral gavage for Aya *vs* intra-BLA infusion for propranolol) and the timing of drug administration relative to memory retrieval [22, 76].

An increase in dendritic spine density has been associated with attenuation of stress-induced impairments in synaptic plasticity. In a chronic stress model using social isolation in non-human primates (*Callithrix jacchus*), Fernandes Pereira et al. (2026) showed that when Aya was administered before and throughout an isolation period, the isolated group exhibited a significant reduction in neuronal volume compared with the family group (control), whereas the Aya-treated group displayed neuronal volumes comparable to those of the family group, suggesting a prophylactic effect of Aya [77]. Interestingly, parallel evidence exists for ketamine, which, despite not being a classical psychedelic, shares with Aya the ability to induce neuroplasticity and modulate fear and stress behavioral outcomes [78–81]. When administered before a stressor, ketamine confers lasting resilience against depressive- and PTSD-like behavior induced by stress in rodents [82, 83], an effect that has been linked to structural plasticity changes, including altered dendritic spine density and morphology in brain regions relevant to memory processing, such as the hippocampus [84, 85]. While ketamine's protective effect is typically studied when given before stress exposure, our findings suggest that Aya may confer a comparable form of protection when given after memory retrieval, disrupting the reconsolidation of an already consolidated aversive memory rather than preventing its initial formation. This raises the possibility that structural plasticity mechanisms similar to those recruited by prophylactic ketamine may also underlie Aya's capacity to weaken aversive fear memories.

The effects of Aya observed here are also comparable with other classic psychedelics. A single dose of psilocybin increased the number of dendritic spines in the medial prefrontal cortex for up to one month [60]. Although we did not determine how long the Aya-induced changes in dendritic spines persisted, we previously reported that the reduction in freezing behavior lasted up to 22 days after memory retrieval and treatment [15]. Thus, long-lasting behavioral changes may be supported by persistent synaptic modifications. In this context, it is worth highlighting that the effects of DMT on dendritic morphology appear to be dose-, treatment regimen-, and sex-dependent. A single high dose (10 mg/kg, i.p.) increased spine density in the prefrontal cortex of adult male rats [52], whereas chronic intermittent administration of a low dose (1 mg/kg) reduced spine density in females but not in males [53]. These findings indicate that dose, treatment regimen, and sex are important variables to consider when interpreting and generalizing the effects of psychedelics on structural plasticity.

### Effects of Aya Treatment on Neurogenesis

New neurons are continuously generated in the DG subgranular zone of mammals, including rats, and the proportion of BrdU-labeled cells expressing DCX is particularly high around 7–10 days after cell birth, decreasing during the following weeks, becoming lower by 30 days in rats [86–89]. The level of adult hippocampal neurogenesis is related to fear memory persistence [90, 91]. For instance, decreased neurogenesis resulted in enhanced long-term potentiation and fear memory persistence [92]. Here, the treatment with Aya either 20 min before or 3 h after retrieval did not change the DCX expression 10 days after treatment. Thus, suggesting that Aya-induced impairment in fear memory reconsolidation possibly does not rely on neurogenesis mechanisms. Accordingly, mature and immature adult-born neurons underlie remote, not recent memory reconsolidation [27]. Additionally, considering that enhanced neurogenesis underlies fear memory extinction, our results further reinforce that our protocol induces reconsolidation instead of extinction [93, 94]. Here, we analyzed DCX, a common marker of immature neurons in the adult hippocampus. Although DCX expression alone does not represent the whole process of adult neurogenesis, it indicates the presence of immature neurons, which are critical for neurogenesis [86].

Classic psychedelics such as DMT and its analogues have been reported to promote neurogenesis [34, 95]. For instance, administration of 2 mg/kg DMT (i.p.) activated the neurogenic niche in the subgranular zone of DG in mice, and repeated administration of the same dose on alternate days for 21 days further increased neurogenesis in this region [34]. Likewise, a single intracerebroventricular injection of 5-MeO-DMT, a structural analogue of DMT, increased neurogenesis and promoted more complex dendritic arborization of DG granule cells 21 days after treatment [95]. At first glance, these findings appear to contrast with our results. However, important methodological differences should be considered, including the dose, treatment regimen, route of administration, species, and, importantly, the timing of drug administration relative to the behavioral manipulation. Here, we administered a single dose of Aya containing 0.24 mg of DMT within a specific reconsolidation time window, making direct comparisons difficult. Moreover, the previous studies evaluated neurogenesis under basal conditions rather than following memory reactivation, whereas our study specifically examined neurogenesis 10 days after retrieval-induced reconsolidation. It is also noteworthy that adult-born granule neurons require several weeks to mature and become functionally integrated into hippocampal circuits, suggesting that changes in neurogenesis are unlikely to contribute directly to the relatively rapid cellular events underlying memory reconsolidation [86]. Consistent with this interpretation, fear-conditioned rats treated with Veh did not differ from naïve animals, supporting the view that reconsolidation itself may not rely on neurogenesis-related mechanisms. Instead, reconsolidation is thought to depend primarily on rapid synaptic remodeling, de novo protein synthesis, and structural plasticity within pre-existing neuronal ensembles rather than on the generation of new neurons [96, 97].

### Methodological Considerations

Although our data clearly shows that Aya treatment enhances structural plasticity, we did not differentiate dendritic spine morphology. Thus, it was not possible to classify spines into subtypes such as thin, stubby, mushroom, and filopodia, which are associated with different stages of spine maturation and synaptic stability and functionality [98, 99]. The absence of this analysis limits the interpretation of whether the observed increase in dendritic spine density reflects structural maturation or further functional synaptic integration. Similarly, the methodologies used in the present study did not allow direct assessment of spine functionality. Therefore, it remains unclear whether the increase in dendritic spine number induced by Aya corresponds to enhanced synaptic efficacy, stabilization of newly formed synapses resulting in the behavioral changes we observe after Aya treatment, or if it is merely transient structural remodeling.

Another limitation here is the fact we assessed DCX alone as a neurogenesis marker. Although DCX is a widely accepted marker of immature neurons commonly used to evaluate adult neurogenesis, its expression alone does not provide the whole scenario of neurogenesis [100].

Evidence showed that chronic and intermittent treatment with DMT affected differently dendritic spine remodeling in males and female rats, promoting a dendritic spine reduction in the V layer of pyramidal neurons of the prefrontal cortex only in females [53]. Even though this finding refers to DMT alone and was evaluated under experimental conditions different from those used in our study, it would be important to investigate the effects of Aya in female subjects.

## Conclusion

Together, the present findings demonstrate that in males, Aya treatment during early and late phases of reconsolidation impairs fear memory reconsolidation, while it also increases dendritic spine density and promotes structural remodeling in brain regions relevant to fear memory processing. These results bring novel insights into the molecular mechanisms underlying Aya's effects on reconsolidation and support future investigation into the development of psychedelic-based therapies for PTSD.

## Data Availability

All data supporting this study are available from the corresponding author upon request.
